# Evaluation of Chatbot Responses to Text-Based Multiple-Choice Questions in Prosthodontic and Restorative Dentistry

**DOI:** 10.3390/dj13070279

**Published:** 2025-06-21

**Authors:** Reinhard Chun Wang Chau, Khaing Myat Thu, Ollie Yiru Yu, Richard Tai-Chiu Hsung, Denny Chon Pei Wang, Manuel Wing Ho Man, John Junwen Wang, Walter Yu Hang Lam

**Affiliations:** 1 Faculty of Dentistry, The University of Hong Kong, Hong Kong 999077, China; rcwchau@hku.hk (R.C.W.C.); khaing@hku.hk (K.M.T.); ollieyu@hku.hk (O.Y.Y.); richardhsung@chuhai.edu.hk (R.T.-C.H.); denny.wang@alumni.ucl.ac.uk (D.C.P.W.); manuelman.restorative@gmail.com (M.W.H.M.); junwen@hku.hk (J.J.W.); 2 Department of Computer Science, Hong Kong Chu Hai College, Hong Kong 999077, China; 3 Musketeers Foundation Institute of Data Science, The University of Hong Kong, Hong Kong 999077, China

**Keywords:** artificial intelligence, deep learning, machine learning, mouth rehabilitation, natural language processing

## Abstract

**Background/Objectives**: This study aims to evaluate the response accuracy and quality of three AI chatbots—GPT-4.0, Claude-2, and Llama-2—in answering multiple-choice questions in prosthodontic and restorative dentistry. **Methods**: A total of 191 text-based multiple-choice questions were selected from the prosthodontic and restorative dentistry sections of the United States Integrated National Board Dental Examination (INBDE) (n = 80) and the United Kingdom Overseas Registration Examination (ORE) (*n* = 111). These questions were inputted into the chatbots, and the AI-generated answers were compared with the official answer keys to determine their accuracy. Additionally, two dental specialists independently evaluated the rationales accompanying each chatbot response for accuracy, relevance, and comprehensiveness, categorizing them into four distinct ratings. Chi-square and post hoc Z-tests with Bonferroni adjustment were used to analyze the responses. The inter-rater reliability for evaluating the quality of the rationale ratings among specialists was assessed using Cohen’s Kappa (κ). **Results**: GPT-4.0 (65.4%; *n* = 125/191) demonstrated a significantly higher proportion of correctly answered multiple-choice questions when compared to Claude-2 (41.9%; *n* = 80/191) (*p* < 0.017) and Llama-2 (26.2%; *n* = 50/191) (*p* < 0.017). Significant differences were observed in the answer accuracy among all of the chatbots (*p* < 0.001). In terms of the rationale quality, GPT-4.0 (58.1%; *n* = 111/191) had a significantly higher proportion of “Correct Answer, Correct Rationale” than Claude-2 (37.2%; *n* = 71/191) (*p* < 0.017) and Llama-2 (24.1%; *n* = 46/191) (*p* < 0.017). Significant differences were observed in the rationale quality among all of the chatbots (*p* < 0.001). The inter-rater reliability was very high (κ = 0.83). **Conclusions**: GPT-4.0 demonstrated the highest accuracy and quality of reasoning in responding to prosthodontic and restorative dentistry questions. This underscores the varying efficacy of AI chatbots within specialized dental contexts.

## 1. Introduction

The advent of artificial intelligence (AI) has revolutionized various sectors, including healthcare and education [1,2], with significant advancements in Generative AI models such as ChatGPT (OpenAI, San Francisco, CA, USA), Claude-2 (Anthropic, San Francisco, CA, USA), and Llama-2 (Meta, Menlo Park, CA, USA). These models are renowned for their ability to generate complex, human-like responses [3,4]. GPT-4.0, a sophisticated multimodal model, excels in natural language understanding and generation, and is utilized in applications ranging from education to technical support, reportedly performing at a level comparable to the top 10% of professional and academic benchmarks [5]. Claude-2 is designed with a focus on safety, featuring a large context window of up to 100,000 tokens and strong reasoning abilities, making it suitable for complex tasks like those in healthcare and education [6]. Llama-2 is an open-source model, optimized for dialogue with an emphasis on safety, offering flexibility for both research and commercial applications [7].

In dentistry, AI chatbots have emerged as promising tools for enhancing patient communication and assisting in clinical decision-making [5,6,7]. These chatbots leverage advanced language models to interpret and generate human-like text, offering potential benefits in patient interactions and educational settings.

Dental licensing examinations, such as the Integrated National Board Dental Examination (INBDE [8]) in the United States (US) and the Overseas Registration Examination (ORE [9]) in the United Kingdom (UK), serve as essential benchmarks for evaluating the knowledge and competency of aspiring dentists. These examinations encompass a broad range of dental disciplines, with prosthodontic and restorative dentistry being the central component of the dental curriculum [10,11]. The integrity and reliability of these exams are paramount, as they ensure that licensed dentists possess the requisite expertise to provide quality daily patient care.

Despite the growing integration of AI in dental education and practice, from diagnostics and treatment planning to treatment provision [12,13], research investigating the proficiency of AI models in dental knowledge remains limited, especially regarding their proficiency in dental knowledge and the rationales they use to reach conclusions in dentistry. A previous study reported that some versions of AI chatbots could pass general licensing examinations (multiple choice) [14]; a few other studies reported that some of the AI chatbots could respond with relatively accurate answers for yes/no and multiple-choice questions regarding pediatric dentistry and endodontics [15,16,17]. Various studies have also reported the results in orthodontics [18]. However, their performance in prosthodontic and restorative dentistry, especially the correctness of their rationales for multiple-choice answers, remains unclear. Understanding the capabilities and limitations of the latest AI models in this context is crucial for educators, practitioners, and policymakers aiming to harness these technologies effectively.

This study aims to bridge this knowledge gap by evaluating the accuracy of three prominent AI chatbots—GPT-4.0, Claude-2, and Llama-2—in answering questions from prosthodontic and restorative dentistry sections of the INBDE and ORE. The null hypothesis posits that there are no significant differences in the correctness of multiple-choice answers and the rationales provided by these AI chatbots. By evaluating the performance of these chatbots, this research seeks to inform the potential role of AI in prosthodontic and restorative dentistry.

## 2. Materials and Methods

### 2.1. Study Design and Question Selection

This study employed a quantitative, comparative design to evaluate the performance of three AI chatbots in answering prosthodontic and restorative dentistry questions from dental licensing exams from the United States and the United Kingdom. These countries collectively represent 34% of the top 100 dental schools, according to the latest Quacquarelli Symonds (QS) ranking for 2024 [19].

A total of 191 multiple-choice questions were obtained from the top-selling INBDE Book [20] on the US online bookstore Amazon [21], and from the two books [22,23] in the ORE series, which are available through major online bookstores, including Book Depository in the UK [24]. These questions encompassed all the available and relevant questions from these sources, including 80 prosthodontic questions from the INBDE [8] and 111 restorative dentistry questions from the ORE [9]. To ensure consistency in the evaluation by the AI chatbots, questions that contained figures or tables were excluded to eliminate additional variables that might require interpretation.

The questions were input by an independent evaluator (RCWC) in the exact format as they appeared in the books between 29 January 2024 and 4 March 2024, with INBDE questions being input from 29 January 2024 to 8 February 2024, and ORE questions from 26 February 2024 to 4 March 2024.

### 2.2. Chatbot Selection and Configuration

Three AI chatbots were selected for evaluation: GPT-4.0 (OpenAI, San Francisco, CA, USA), Claude-2 (Anthropic, San Francisco, CA, USA), and Llama-2 (Meta, Menlo Park, CA, USA). These chatbots were selected due to their prominence and widespread recognition as leading models during the study period [25,26]. Each chatbot was accessed using default settings through a paid application program interface (API) service to ensure consistency in response generation [27]. The chatbots were instructed to provide answers with rationale, and no additional prompts were instructed to minimize variability. Each examination was answered by each AI chatbot in a separate chat session.

### 2.3. Evaluation of the Accuracy of AI-Generated Multiple-Choice Answers

The responses generated by the AI chatbots were collected and evaluated. The accuracy of the responses was determined by comparing them with the official multiple-choice answer keys of the INBDE and ORE. Additionally, each response was accompanied by a rationale generated by the respective AI chatbot, which was subsequently evaluated by a panel of two calibrated UK-trained dental specialists, A and B (DCPW and MWHM).

### 2.4. Evaluation of the Quality of Rationale Accompanying the Multiple-Choice Answers

The panel employed a standardized rubric to evaluate each rationale as Correct, Partly correct, or Wrong based on the following criteria [28,29]:

- Accuracy: Whether the rationale is correct.
- Relevance and comprehensiveness: Whether the rationale directly relates to the question and fully addresses all necessary elements to support the answer.

Responses were categorized and rated as follows:

- Correct Answer, Correct Rationale: The AI chatbot answered accurately and considered all factors correctly, making its response both relevant and comprehensive.
- Correct Answer, Partly Correct/Wrong Rationale: The AI chatbot answered accurately but did not consider all factors correctly, making its rationale either partly or completely irrelevant and/or incomplete.
- Wrong Answer, Correct Rationale: The AI chatbot answered incorrectly but considered all factors correctly, making its rationale relevant and comprehensive.
- Wrong Answer, Partly Correct/Wrong Rationale: The AI chatbot answered incorrectly and did not consider all factors correctly, making its rationale either partly or completely irrelevant and/or incomplete.

### 2.5. Statistical Analysis

Data were analyzed using IBM SPSS Statistics Version 29 (IBM, Armonk, NY, USA). The performance of the three AI chatbots—GPT-4.0, Claude-2, and Llama-2—was evaluated regarding answer accuracy and rationale quality. Descriptive statistics were used to summarize the performance metrics of each AI chatbot.

Chi-square tests of homogeneity were used to determine whether there were significant differences in the distribution of answer accuracy and rationale quality ratings across the three AI chatbots. Post hoc pairwise Z-tests with Bonferroni adjustments were performed to identify differences between chatbots. The significance level was adjusted to α = 0.017 (0.05/3) to control for Type I error across the three comparisons.

Inter-rater reliability between Specialist A and B was computed using Cohen’s Kappa (κ) to ensure consistency and agreement in evaluating rationale quality ratings.

## 3. Results

### 3.1. Accuracy of AI-Generated Multiple-Choice Answers

The Chi-square tests of homogeneity revealed significant differences in the answer accuracy among the chatbots, χ^2^(2, *N* = 573) = 60.416, *p* < 0.001 (Table 1).

GPT-4.0 demonstrated the highest accuracy, with 65.4% (*n* = 125/191) of its responses classified as “Correct Answer” and 34.6% (*n* = 66/191) as “Wrong Answer”. GPT-4.0’s accuracy is significantly higher than that of Claude-2 and Llama-2 (*p* < 0.017).

Claude-2 exhibited a lower accuracy, with 41.9% (*n* = 80/191) of its responses rated as “Correct Answer” and 58.1% (*n* = 111/191) as “Wrong Answer”. Claude-2’s accuracy is significantly lower compared to that of GPT-4.0 but higher than that of Llama-2 (*p* < 0.017).

Llama-2 showed the lowest accuracy, with only 26.2% (*n* = 50/191) of its responses classified as “Correct Answer” and 73.8% (*n* = 141/191) as “Wrong Answer”. Llama-2’s performance is significantly poorer compared to both GPT-4.0 and Claude-2 (*p* < 0.017) in the studied parameter.

### 3.2. Quality of Rationale Accompanying the Multiple-Choice Answers

The Chi-square tests of homogeneity indicated significant differences in the rationale quality ratings among the chatbots, χ^2^(6, *N* = 573) = 71.776, *p* < 0.001 (Table 2).

GPT-4.0 demonstrated the highest proficiency in delivering high-quality rationales, having 58.1% (*n* = 111/191) of its responses rated “Correct Answer, Correct Rationale”. There were significant differences between this category of GPT-4.0 and those of Claude-2 (*p* < 0.017) and Llama-2 (*p* < 0.017). This showcased GPT-4.0’s ability to provide accurate answers and quality rationales.

Claude-2 demonstrated a lower proficiency in delivering high-quality rationales, having 37.2% (*n* = 71/191) of its responses rated “Correct Answer, Correct Rationale”. In addition, there was a significant difference between Claude-2 and Llama-2 in “Correct Answer, Correct Rationale” (*p* < 0.017), indicating that while Claude-2 did not perform as well as GPT-4.0, it still had a higher ability to provide accurate answers and quality rationales compared to that of Llama-2.

Llama-2 exhibited the lowest performance in providing high-quality rationales, having only 24.1% (*n* = 46/191) of its responses rated “Correct Answer, Correct Rationale”. It also had 63.4% (*n* = 121/191) of its responses rated under the “Wrong Answer, Partly Correct/Wrong Rationale” category, indicating a significantly worse performance (*p* < 0.017) than the other chatbots. This highlights Llama-2’s need for improvement in generating accurate answers and quality rationales.

**Figure 1:**
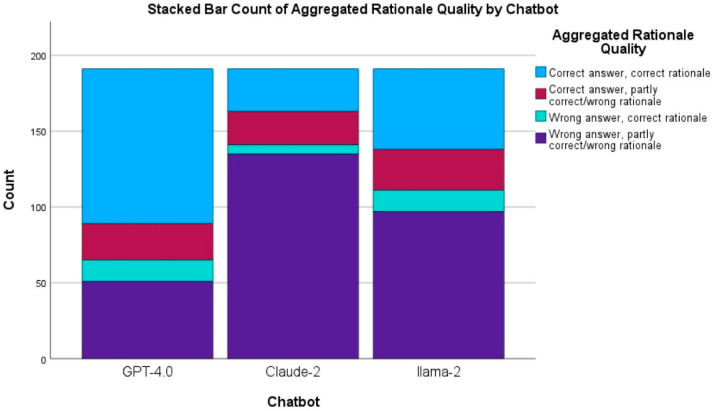
Stacked bar count of chatbot by overall rationale scores.

The results are illustrated in Figure 1.

### 3.3. Inter-Rater Reliability

The overall inter-rater reliability (Cohen’s Kappa, κ) between Specialist A and Specialist B was computed, resulting in a value of κ = 0.83, indicating almost perfect (range 0.81–0.99) agreement [30]. For GPT-4.0, the Cohen’s Kappa (κ) between the two specialists was 0.96, indicating almost perfect agreement. For Claude-2, the Cohen’s Kappa (κ) between the two specialists was 0.82, indicating almost perfect agreement [30]. For Llama-2, the Cohen’s Kappa (κ) between the two specialists was 0.72, indicating substantial agreement. These Cohen’s Kappa values affirmed the reliability of the ratings of the chatbots’ rationale quality.

## 4. Discussion

AI has transformative potential across various domains of dentistry. In diagnostics, AI algorithms can enhance the accuracy of radiographic interpretations and disease screening [31,32], enabling the early detection of possible oral pathologies that may be overlooked by the human eye. Treatment planning can be personalized through AI-driven predictive models that anticipate patient-specific outcomes, optimizing restorative procedures [12,33]. The rapid development of AI chatbots presents significant promise for medical education and patient management, particularly in providing accessible learning resources and enhancing patient communication. The accuracy and quality of the generated answers are of the utmost importance for these applications. As the consistency of chatbots in answering prosthodontic and restorative dentistry questions has been investigated [34,35], this study primarily aimed to evaluate the efficacy of three prominent AI chatbots—GPT-4.0, Claude-2, and Llama-2—in accurately addressing prosthodontic and restorative dentistry questions from dental licensing exams. Via a comparative evaluation of the accuracy and rationale quality of the AI chatbots in addressing prosthodontic and restorative dentistry multiple-choice questions from two dental licensing exams, this study aimed to shed light on the complexity involved in selecting a multiple-choice answer and understanding the rationale behind it.

The null hypothesis that there were no significant differences in the correctness of the multiple-choice answers and the rationales provided by these AI chatbots has been rejected, as a performance difference has been observed. GPT-4.0 exhibited the highest accuracy in answering the multiple-choice questions among the evaluated chatbots, correctly answering approximately 74% of NBDE and 60% of ORE questions, making it a likely preference for real-life applications. While these figures demonstrate a promising capability, particularly for GPT-4.0, the performance of Claude-2 and Llama-2 was significantly lower, with Llama-2 showing significant limitations in accuracy. This suggests that although advanced language models like GPT-4.0 are approaching the proficiency level of a qualified dentist and can serve as supplementary educational and clinical tools, there remains substantial room for improvement, especially in the accuracy and quality of responses from other advanced language models.

The evaluation of rationales provided by the chatbots revealed a similar trend. The findings indicate that GPT-4.0 significantly outperforms Claude-2 and Llama-2 in providing high-quality rationales for multiple-choice questions for prosthodontic and restorative dentistry. These differences are statistically significant, with a *p*-value of less than 0.001 and an effect size of 0.250. The ability to generate correct answers along with high-quality explanatory content is crucial for educational purposes and effective patient communication [36]. Furthermore, the results of this study align with previous research investigating the performance of AI chatbots across various areas of dentistry [15,17,37].

Several factors may contribute to the varying performance levels observed among the chatbots. The underlying training data, model architecture, and fine-tuning processes are pivotal in determining a chatbot’s proficiency in specialized domains like prosthodontic and restorative dentistry. GPT-4.0’s superior performance could be attributed to its more extensive training data and advanced algorithms, which enhance its contextual understanding and reasoning capabilities [38,39]. On the other hand, it is crucial to note that while GPT-4.0 performed better than its counterparts, it is still not perfect and is subject to errors and inaccuracies [16,26].

This study has limitations. The methodology did not assess response consistency across repeated trials, specific question types, or chatbot training data, thereby limiting insights into their full capabilities. Given the rapid evolution of AI chatbots, new models or updates may have emerged since the study’s completion (March 2024), necessitating updated research to remain relevant. Although the consistency of chatbots were not investigated in this study—areas addressed by previous research [34,35]—future studies should examine the consistency of new chatbot models alongside their accuracy and underlying rationale. This is especially important because new models are continually emerging, and there may be a lack of studies investigating their consistency.

AI-driven educational tools can revolutionize oral health education by offering interactive simulations and personalized feedback, potentially improving patient outcomes [40]. However, challenges like data privacy, bias mitigation, and AI consistency must be addressed [33,41]. Future research should include a broader range of questions, additional chatbot models, and response consistency assessments to identify improvement areas. Qualitative analyses should also evaluate the suitability of AI-generated information for patient communication.

## 5. Conclusions

This study provides a comparative evaluation of the accuracy and quality of AI chatbots in addressing prosthodontic and restorative dentistry questions from dental licensing examinations, based on a single round of evaluation. GPT-4.0 demonstrated the highest accuracy and rationale quality among the evaluated models, highlighting its potential as a valuable educational tool in the near future. Continued advancements in AI technology, combined with rigorous evaluation frameworks that include assessments of the response consistency, will be essential in realizing the full potential of chatbots in the dental field. AI chatbots may make mistakes, and it is imperative to verify important information.

## Figures and Tables

**Table 1:** Distribution of answer accuracy across chatbots.

CHATBOT	CORRECT ANSWER	WRONG ANSWER
GPT-4.0	125/191 (65.4%) _a_	66/191 (34.6%) _a_
CLAUDE-2	80/191 (41.9%) _b_	111/191 (58.1%) _b_
LLAMA-2	50/191 (26.2%) _c_	141/191 (73.8%) _c_

Each subscript letter denotes a subset of accuracy rating categories whose row proportions do not differ significantly from each other at the 0.001 level.

**Table 2:** Distribution of rationale quality across chatbots.

CHATBOT	CORRECT ANSWER		WRONG ANSWER	
	Correct Rationale	Partly Correct/Wrong Rationale	Correct Rationale	Partly Correct/Wrong Rationale
GPT-4.0	111/191 (58.1%) _a_	14/191 (7.3%) _a_	22/191 (11.5%) _a_	44/191 (23.0%) _a_
CLAUDE-2	71/191 (37.2%) _b_	10/191 (5.2%) _a,b_	29/191 (15.2%) _a_	81/191 (42.4%) _b_
LLAMA-2	46/191 (24.1%) _c_	4/191 (2.1%) _b_	20/191 (10.5%) _a_	121/191 (63.4%) _c_

Each subscript letter denotes a subset of accuracy rating categories whose row proportions do not differ significantly from each other at the 0.017 level.

## Data Availability

The data presented in this study are available on request from the corresponding author due to copyright restrictions.

## Abbreviations

The following abbreviations are used in this manuscript:

NBDE	United States National Board Dental Examination
ORE	Overseas Registration Examination
AI	Artificial Intelligence
US	United States
UK	United Kingdom
QS	Quacquarelli Symonds
OHI	Oral Health Instructions
